# Hospital burden and clinical features associated with admission among confirmed measles cases during the 2025-2026 outbreak at a tertiary care hospital in Mexico

**DOI:** 10.1016/j.ijregi.2026.100965

**Published:** 2026-07-31

**Authors:** Luis Eduardo del Moral-Trinidad, Paola Elizabeth Trejo-González, Christian Alberto Acosta-Aguilar, Brian Rafael Rubio-Mora, Karla Isis Avilés-Martínez, Melva Guadalupe Herrera-Godina, Martín Guerrero-Becerra, Rodrigo Escobedo-Sánchez, Esteban González-Díaz, Gilberto Silva-Bañuelos

**Affiliations:** 1 Doctorado en Ciencias de la Salud Pública, Centro Universitario de Ciencias de la Salud, Universidad de Guadalajara, Guadalajara, Mexico; 2 Unidad de Medicina Preventiva y Vigilancia Epidemiológica, Hospital Civil de Guadalajara “Fray Antonio Alcalde”, Guadalajara, Mexico; 3 Urgencias Adultos, Hospital Civil de Guadalajara “Fray Antonio Alcalde”, Guadalajara, Mexico; 4 Urgencias Pediatría, Hospital Civil de Guadalajara “Fray Antonio Alcalde”, Guadalajara, Mexico; 5 Departamento de Salud Pública, Centro Universitario de Ciencias de la Salud, Universidad de Guadalajara, Guadalajara, Mexico; 6 Centro de Investigaciones en Sistemas de Información Geográfica en Salud, Departamento de Salud Pública, Centro Universitario de Ciencias de la Salud, Universidad de Guadalajara, Jalisco, Mexico; 7 Infectología Pediátrica, Hospital Civil de Guadalajara “Fray Antonio Alcalde”, Guadalajara, Mexico; 8 Infectología Adultos, Hospital Civil de Guadalajara “Fray Antonio Alcalde”, Guadalajara, Mexico; 9 Instituto de Patología Infecciosa y Experimental “Francisco Ruiz Sánchez”, Centro Universitario de Ciencias de la Salud, Universidad de Guadalajara, Guadalajara 44430, Mexico

**Keywords:** Measles, Hospitalization, Outbreak, Vaccination, Mexico

## Abstract

**Objectives:**

Measles has re-emerged as a public health concern despite effective vaccination. This study describes the clinical and epidemiological characteristics associated with hospital admission among confirmed measles cases attended at a tertiary referral hospital in Jalisco, Mexico, during the 2025-2026 outbreak.

**Methods:**

We conducted an observational, hospital-based cohort study of confirmed measles cases attended from November 2025 to May 2026. Congenital measles cases and records with missing hospitalization status were excluded. Data were obtained from clinical records and surveillance systems. Univariable logistic regression and two multivariable models were fitted: an epidemiological model excluding respiratory severity markers and a clinical presentation model including oxygen saturation and respiratory rate.

**Results:**

Among 609 confirmed cases, 186/609 patients (30.5%) were hospitalized. Most were pediatric patients (460/609 [75.5%]), and 489/609 (80.3%) were eligible but unvaccinated. Among hospitalized patients, pneumonia was the most frequent complication (51/186 [27.4%]), and 107/186 (57.5%) required supplemental oxygen. In the epidemiological model, each additional day from symptom onset to medical care was associated with hospitalization (adjusted odds ratio [aOR] 1.14 per day; 95% confidence interval [CI] 1.04-1.25), lower oxygen saturation (aOR 3.51 per 5-percentage-point decrease; 95% CI 2.53-4.98), and higher respiratory rate (aOR 1.27 per 5 breaths/min increase; 95% CI 1.05-1.54).

**Conclusion:**

Hospital admission was common and was mainly characterized by delayed care and objective respiratory compromise.

## Highlights

• We analyzed 609 confirmed measles cases in Mexico.
• Hospital admission occurred in 186 patients.
• Pneumonia was the leading hospitalized complication.
• Respiratory compromise characterized hospital admission.
• Vaccinated hospitalized cases were described separately.

## Introduction

Measles is one of the most contagious viral diseases affecting humans and remains a major public health concern despite the availability of a safe and highly effective vaccine. Its high transmissibility, with an estimated basic reproductive number ranging from 12 to 18, facilitates rapid spread in susceptible populations, particularly where vaccination coverage is insufficient. Although global immunization efforts have markedly reduced measles-related mortality, recent years have seen a renewed increase in outbreaks, driven by declining vaccination coverage, unequal access to health services, vaccine misinformation, and the accumulation of susceptible individuals [[1], [2], [3]].

The coronavirus disease 2019 pandemic further disrupted routine immunization programs worldwide, contributing to delays in vaccination campaigns, reduced access to preventive services, and logistical barriers to vaccine delivery. These interruptions increased the risk of the resurgence of vaccine-preventable diseases, including measles. Recent global surveillance data have shown a rise in suspected and confirmed measles cases across multiple regions, underscoring the difficulty of maintaining the herd immunity required to interrupt transmission, which depends on vaccination coverage above 95% with two doses [[4], [5], [6], [7], [8], [9]].

In the Region of the Americas, measles outbreaks have re-emerged despite previous elimination efforts. Mexico interrupted endemic measles transmission in 1997, after decades of vaccination campaigns and strengthened epidemiological surveillance. However, sporadic imported cases and subsequent outbreaks have been documented in recent years. Historically, the largest recent epidemic in Mexico occurred in 1989–1990, and the last autochthonous case was reported in 1995; nevertheless, a new outbreak was documented in 2020, and more recent surveillance data indicate renewed transmission in several states [[10], [11], [12]].

Jalisco has been one of the most affected states during the recent measles outbreak in Mexico. According to state surveillance data, by March 2026, Jalisco had accumulated 3386 confirmed measles cases, an incidence rate of 38.53 per 100,000 population, and three deaths, ranking second nationally both in incidence and in the number of confirmed cases. Within the state, transmission was concentrated in municipalities of the Guadalajara metropolitan area, with Tlaquepaque reporting the highest number of cases. During the same outbreak period, our hospital served as a regional referral center for suspected and confirmed measles cases, evaluating approximately 900 suspected cases through clinical care and epidemiological surveillance pathways. This local burden provided a unique opportunity to describe the hospital-based clinical spectrum and outcomes of measles during a contemporary outbreak [13].

Beyond its epidemiological impact, measles can impose a substantial burden on hospital services. The disease may lead to complications such as pneumonia, otitis media, encephalitis, and secondary bacterial infections, particularly among young children, unvaccinated adults, malnourished individuals, pregnant women, and immunocompromised patients. Severe cases may require hospital isolation, close monitoring, oxygen therapy, antibiotics for suspected bacterial complications, ventilatory support, or admission to an intensive care unit [2,3,14,15].

Despite the public health relevance of recent measles outbreaks, clinical evidence from hospital-based cohorts in Mexico and Latin America remains limited. Most available reports have focused on population-level surveillance, incidence, and outbreak dynamics, whereas fewer studies have described the clinical spectrum, hospital outcomes, and features associated with hospitalization during recent outbreaks. Therefore, this study aimed to describe the clinical and epidemiological characteristics associated with hospital admission among confirmed measles cases treated at a tertiary referral hospital in Jalisco, Mexico, during the 2025-2026 outbreak.

## Methods

### Study design and setting

We conducted an observational, analytical, hospital-based cohort study of confirmed measles cases attended during a measles outbreak in Mexico. The study was performed at the Antiguo Hospital Civil de Guadalajara “Fray Antonio Alcalde,” a tertiary referral hospital that provides care to pediatric and adult patients. During the outbreak period, the hospital served as a regional referral center and evaluated approximately 900 suspected measles cases. The study period covered confirmed cases identified during the outbreak surveillance period from November 2025 to May 2026.

We reported this study in accordance with the Strengthening the Reporting of Observational Studies in Epidemiology guidelines.

### Participants

Eligible participants were patients with confirmed measles who were attended at the study hospital during the study period. Cases were classified as confirmed according to laboratory evidence or epidemiological linkage documented in the institutional clinical records and epidemiological surveillance system.

For the main analysis, congenital measles cases were excluded because of differences in the transmission mechanism and clinical course, which limit comparability with cases acquired through respiratory transmission. Records with missing hospitalization status were also excluded from the analytical cohort.

### Data sources and data collection

Data were obtained from clinical records, hospital documentation, laboratory results, and epidemiological surveillance records. A standardized data collection form was used to extract sociodemographic, epidemiological, clinical, laboratory-confirmed, management, and outcome variables.

### Variables

Confirmed measles cases included laboratory-confirmed and epidemiologically linked cases according to the surveillance criteria. Laboratory confirmation was based on the detection of measles virus by reverse-transcription polymerase chain reaction, positive measles-specific immunoglobulin M serology, or serological evidence compatible with recent infection in paired samples when available. Epidemiologically linked cases were defined as clinically compatible cases with documented exposure or linkage to a confirmed case during the outbreak, as recorded in the epidemiological surveillance system.

#### Outcome variable

The primary outcome was hospitalization, defined as documented hospital admission during the measles episode. Patients were classified as hospitalized or non-hospitalized according to clinical and hospital records. Hospital admission decisions were made by the treating clinical team according to the institutional clinical criteria. Admission was generally considered for patients with respiratory compromise or hypoxemia, suspected or confirmed complications, a need for oxygen therapy or close monitoring, age-related or host-related risk factors, an inability to ensure safe outpatient follow-up, or a need for hospital-based isolation. No single criterion was used as an exclusive indication for hospitalization; rather, admission reflected the overall clinical assessment during the outbreak.

Secondary clinical outcomes included pneumonia, encephalitis, supplemental oxygen requirement, high-flow nasal cannula use, invasive mechanical ventilation, intensive care unit (ICU) admission, length of hospital stay, and death. Pneumonia was defined according to clinical documentation and/or radiological findings recorded in the medical record. Systemic antibiotic use was recorded when prescribed by the treating team and generally reflected suspected bacterial coinfection or secondary bacterial pneumonia based on clinical, radiological, or laboratory criteria; microbiological confirmation was not required for the initiation of antibiotics.

#### Independent variables

Sociodemographic variables included age, sex, municipality of residence, and age stratum. Age was analyzed as a continuous variable and was categorized into predefined age groups: <1, 1-4, 5-14, 15-29, 30-49, and ≥50 years. Age stratum was defined as pediatric (<18 years) or adult (≥18 years).

Epidemiological variables included vaccination status, travel in the previous 2 weeks, contact or cohabitation with a confirmed case, and final case classification. Vaccination eligibility and status were derived from age and documented vaccination history and categorized as not eligible for routine vaccination (<1 year), eligible and unvaccinated, or eligible with any documented vaccination. Final case classification was categorized as laboratory-confirmed or epidemiologically linked.

Time from symptom onset to medical care was defined as the number of days between symptom onset and the first documented emergency department evaluation at the study hospital. Symptom onset was primarily defined as the date of rash onset when available. When rash onset was not documented, the earliest recorded date of fever or another measles-compatible symptom was used, but only among cases that had already met the confirmation criteria through laboratory evidence or epidemiological linkage. Fever alone was not used to define a suspected measles case; rather, it was used only to assign the symptom onset date when rash onset was unavailable in confirmed cases. Clinical variables included comorbidities, immunosuppression, fever, cough, coryza, conjunctivitis, rash, Koplik spots, oxygen saturation, respiratory rate, heart rate, temperature, and quick Sequential Organ Failure Assessment (qSOFA) at presentation. Cough and coryza were recorded as separate clinical variables because they were captured independently in the clinical and epidemiological surveillance records. Coryza referred to nasal catarrhal symptoms, including rhinorrhea or nasal congestion.

A global comorbidity count was calculated from documented chronic conditions. Treatment and hospital course variables included vitamin A administration, systemic antibiotic use, antiviral use, maximum respiratory support, ICU admission, hospital length of stay, and death. Maximum respiratory support was categorized as none, conventional oxygen therapy, high-flow nasal cannula, or mechanical ventilation.

### Bias

Several measures were taken to reduce information bias. Data were extracted using a structured form, and variables were defined *a priori*. Laboratory-confirmed and epidemiologically linked cases were identified according to surveillance records. Cases with congenital measles were excluded from the main analysis to reduce clinical heterogeneity. Missing values were reported when present, and complete-case analysis was used for regression models.

Because this was a hospital-based study, the findings should be interpreted as associations among patients attended at a tertiary hospital and not as population-level estimates of measles incidence or risk.

### Study size

The study used a consecutive census of confirmed measles cases that were attended during the outbreak period. No formal sample size calculation was performed because all eligible cases during the study period were included.

### Statistical analysis

Continuous variables were summarized using medians and interquartile ranges (IQRs). Categorical variables were summarized as frequencies and percentages. Clinical and epidemiological characteristics were compared between hospitalized and non-hospitalized patients. Continuous variables were compared using the Wilcoxon rank-sum test; categorical variables were compared using chi-square or Fisher’s exact tests, as appropriate.

Univariable logistic regression was used to estimate crude odds ratios and 95% confidence intervals (CIs) for hospitalization. Cough was not included in the univariable regression analysis because all hospitalized patients had cough, resulting in quasi-complete separation and unstable estimates.

Two complementary multivariable logistic regression models were fitted. First, an epidemiological model was constructed to evaluate baseline demographic and epidemiological features associated with hospitalization while avoiding adjustment for potential clinical mediators of severity. This model included age, sex, vaccination eligibility/status, contact with a confirmed case, and days from symptom onset to medical care. Second, a clinical model was fitted to identify clinical markers at presentation associated with hospitalization. This model included age, sex, vaccination eligibility/status, contact with a confirmed case, oxygen saturation at presentation, and respiratory rate at presentation. Because oxygen saturation and respiratory rate may have informed admission decisions, the clinical model should be interpreted as describing admission-associated markers rather than independent causal determinants of hospitalization.

Oxygen saturation was modeled per 5-percentage-point decrease, and respiratory rate was modeled per 5 breaths/min increase to improve clinical interpretability. Adjusted odds ratios (aORs) and 95% CIs were reported for both multivariable models.

Collinearity among predictors was evaluated using variance inflation factors. Model calibration was assessed using the Hosmer–Lemeshow goodness-of-fit test. Discrimination was evaluated using the area under the receiver operating characteristic curve (AUC). Model explanatory performance was described using Nagelkerke’s *R*². A two-sided *P*-value <0.05 was considered statistically significant. All statistical analyses were performed using R statistical software. Missing data were handled using complete-case analysis for regression models. The number of observations included in each model was reported because variables, such as days from symptom onset to medical care and body mass index (BMI), had incomplete data. Analyses involving variables with substantial missingness were interpreted cautiously.

### Sensitivity and stratified analyses

Several sensitivity analyses were performed to evaluate the robustness of the findings. First, an alternative clinical model replaced respiratory rate with qSOFA to evaluate whether the findings were consistent when using a composite clinical severity score. Second, the clinical model was repeated among laboratory-confirmed cases only. Third, exploratory age-stratified analyses were performed among pediatric and adult patients. Because the adult subgroup had fewer hospitalization events, the adult-only model used a more parsimonious covariate structure. The interaction between age stratum and oxygen saturation was assessed using a likelihood-ratio test.

Anthropometric variables were also evaluated in exploratory sensitivity analyses. BMI was calculated when valid weight and height were available; height values recorded as 0 were treated as missing. Because BMI interpretation differs by age and requires age- and sex-specific references in children and adolescents, BMI was not included in the primary models. Instead, BMI was evaluated as a continuous variable in exploratory sensitivity analyses among patients with valid anthropometric data.

### Ethical considerations

The study was conducted using clinical and epidemiological records and did not involve any intervention or modification of patient care. Data were analyzed in a de-identified database and were used exclusively for research purposes.

The study protocol was reviewed and approved by the Comité de Ética en Investigación of the Antiguo Hospital Civil de Guadalajara “Fray Antonio Alcalde” under approval number CEI 084/26 on April 14, 2026. The approved protocol was titled “Características clínicas, epidemiológicas y factores asociados a desenlaces adversos en pacientes con sarampión atendidos en el Antiguo Hospital Civil de Guadalajara ‘Fray Antonio Alcalde,’ 2025–2026”.

Given the observational nature of the study, the use of secondary clinical and surveillance data, and the absence of direct patient intervention, a waiver of informed consent was approved by the ethics committee.

## Results

### Study population

**Figure 1:**
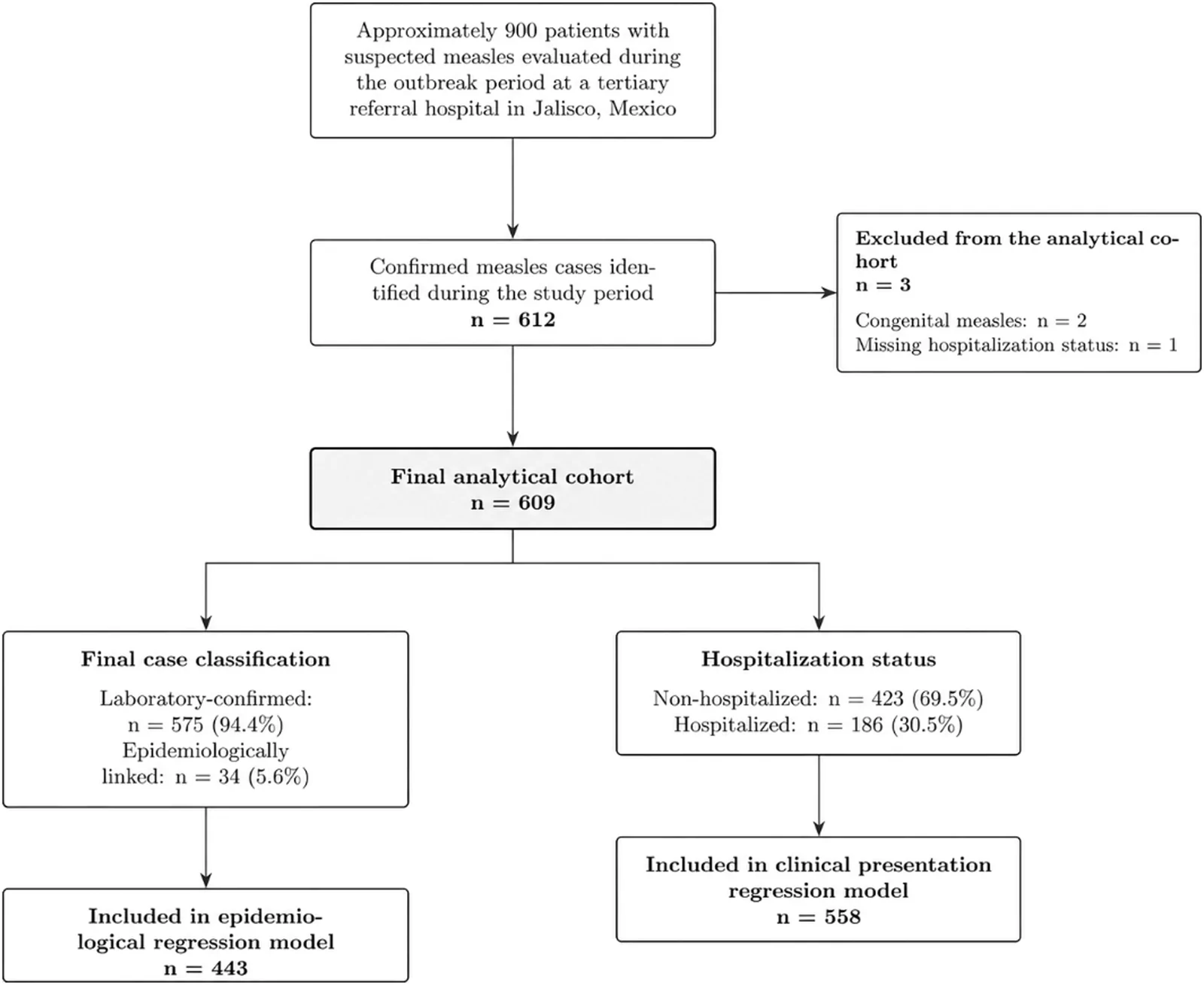
Study flow diagram of confirmed measles cases included in the analytical cohort.

During the study period, approximately 900 patients with suspected measles were evaluated at the hospital. Of these, 612 met the criteria for confirmed measles. After exclusion of two congenital measles cases and one record with missing hospitalization status, 609 confirmed cases were included in the final analytical cohort. Among the confirmed cases, 575 were laboratory-confirmed and 34 were epidemiologically linked (Figure 1). Of these, 443/609 (72.7%) were included in the epidemiological regression model and 558/609 (91.6%) were included in the clinical presentation model, according to variable availability.

### Clinical and epidemiological characteristics according to hospitalization status

Most cases were pediatric patients (460/609 [75.5%]), and the median age of the cohort was 9 years (IQR, 3-17). The age distribution differed by hospitalization status, with hospitalized patients being younger than non-hospitalized patients (8 years [IQR, 2-16] vs 10 years [IQR, 4-18]; *P* = 0.017). Children younger than 1 year represented 61/609 (10.0%) of the cohort and accounted for 26/186 (14.0%) of hospitalized cases.

Most cases were eligible but unvaccinated (488/609 [80.3%]), whereas 58/609 (9.5%) were younger than 1 year and therefore not eligible for the routine vaccination schedule, and 62/609 (10.2%) had documentation of at least one vaccine dose.

Although the median time from symptom onset to seeking medical care was 4 days in both hospitalized and non-hospitalized patients, hospitalized patients had a wider and higher interquartile range (4 days [IQR, 3-6] vs 4 days [IQR, 2.5-5]; *P* = 0.002), suggesting a distribution that shifted toward longer delays. Comorbidities were uncommon: 97.9% of patients had no documented comorbid conditions, and immunosuppression was documented in only 0.5% of cases.

Fever was present in all cases, while cough, coryza, conjunctivitis, rash, and Koplik spots were frequent clinical manifestations. Rash was also nearly universal, occurring in 61/62 (98.4%) patients with any documented measles-containing vaccine dose and in 539/546 (98.7%) patients with no documented routine vaccination or who were not eligible for routine vaccination. Compared with non-hospitalized patients, hospitalized patients more often had conjunctivitis (79.6% vs 67.1%; *P* = 0.002) and Koplik spots (67.7% vs 55.3%; *P* = 0.005). Hospitalized patients also had lower oxygen saturation at admission (94% [IQR, 90-96] vs 96% [IQR, 94-97]; *P* < 0.001) and a higher respiratory rate (24 [IQR, 20-31] vs 21 [IQR, 18-25]; *P* < 0.001). qSOFA scores were also higher among hospitalized patients (*P* < 0.001) (Table 1).

**Table 1:** Clinical and epidemiological characteristics of measles cases according to hospitalization status.

Characteristics	Overall, N = 609	Non-hospitalized, N = 423	Hospitalized, N = 186	*P*-value
Age, years, median (IQR)	9 (3.0-17.0)	10 (4.0-18.0)	8 (2.0-16.0)	0.017
Age group, n (%)				0.039
<1 year	61 (10.0%)	35 (8.3%)	26 (14.0%)	
1-4 years	123 (20.2%)	84 (19.9%)	39 (21.0%)	
5-14 years	228 (37.4%)	162 (38.3%)	66 (35.5%)	
15-29 years	151 (24.8%)	102 (24.1%)	49 (26.3%)	
30-49 years	43 (7.1%)	37 (8.7%)	6 (3.2%)	
≥50 years	3 (0.5%)	3 (0.7%)	0 (0.0%)	
Age stratum, n (%)				0.219
Pediatric	460 (75.5%)	313 (74.0%)	147 (79.0%)	
Adult	149 (24.5%)	110 (26.0%)	39 (21.0%)	
Sex, n (%)				0.399
Female	308 (50.7%)	209 (49.4%)	99 (53.5%)	
Male	301 (49.3%)	214 (50.6%)	86 (46.5%)	
Vaccination eligibility/status, n (%)				0.051
Eligible, unvaccinated	488 (80.3%)	337 (79.9%)	151 (81.2%)	
Not eligible (<1 year)	58 (9.5%)	35 (8.3%)	23 (12.4%)	
Eligible, any vaccination	62 (10.2%)	50 (11.8%)	12 (6.5%)	
Missing	1	1	0	
Travel in the previous 2 weeks, n (%)				0.366
No	592 (97.2%)	409 (96.7%)	183 (98.4%)	
Yes	17 (2.8%)	14 (3.3%)	3 (1.6%)	
Contact with a positive case, n (%)				0.088
No	458 (75.2%)	327 (77.3%)	131 (70.4%)	
Yes	151 (24.8%)	96 (22.7%)	55 (29.6%)	
Final case classification, n (%)				0.735
Laboratory-confirmed	575 (94.4%)	398 (94.1%)	177 (95.2%)	
Epidemiologically linked	34 (5.6%)	25 (5.9%)	9 (4.8%)	
Days from symptom onset to medical care, median (IQR)	4 (3.0-5.0)	4 (2.5-5.0)	4 (3.0-6.0)	0.002
Missing	165	115	50	
Immunosuppression, n (%)				>0.999
No	606 (99.5%)	421 (99.5%)	185 (99.5%)	
Yes	3 (0.5%)	2 (0.5%)	1 (0.5%)	
Number of comorbidities, n (%)				0.563
0	596 (97.9%)	415 (98.1%)	181 (97.3%)	
1	12 (2.0%)	7 (1.7%)	5 (2.7%)	
2	1 (0.2%)	1 (0.2%)	0 (0.0%)	
Diabetes, n (%)				0.672
No	608 (99.8%)	423 (100.0%)	185 (99.5%)	
Yes	1 (0.2%)	0 (0.0%)	1 (0.5%)	
Asthma, n (%)				0.584
No	600 (98.5%)	418 (98.8%)	182 (97.8%)	
Yes	9 (1.5%)	5 (1.2%)	4 (2.2%)	
Chronic kidney disease, n (%)				>0.999
No	608 (99.8%)	422 (99.8%)	186 (100.0%)	
Yes	1 (0.2%)	1 (0.2%)	0 (0.0%)	
Fever, n (%)				
No	0 (0.0%)	0 (0.0%)	0 (0.0%)	
Yes	609 (100.0%)	423 (100.0%)	186 (100.0%)	
Cough, n (%)				0.009
No	18 (3.0%)	18 (4.3%)	0 (0.0%)	
Yes	589 (97.0%)	403 (95.7%)	186 (100.0%)	
Missing	2	2	0	
Coryza, n (%)				0.559
No	108 (17.8%)	78 (18.5%)	30 (16.1%)	
Yes	500 (82.2%)	344 (81.5%)	156 (83.9%)	
Missing	1	1	0	
Conjunctivitis, n (%)				0.002
No	177 (29.1%)	139 (32.9%)	38 (20.4%)	
Yes	431 (70.9%)	283 (67.1%)	148 (79.6%)	
Missing	1	1	0	
Rash, n (%)				0.466
No	8 (1.3%)	7 (1.7%)	1 (0.5%)	
Yes	601 (98.7%)	416 (98.3%)	185 (99.5%)	
Koplik spots, n (%)				0.005
No	249 (40.9%)	189 (44.7%)	60 (32.3%)	
Yes	360 (59.1%)	234 (55.3%)	126 (67.7%)	
Oxygen saturation at admission, %, median (IQR)	95 (93.0-97.0)	96 (94.0-97.0)	94 (90.0-96.0)	<0.001
Missing	47	44	3	
Respiratory rate at admission, median (IQR)	22 (19.0-27.0)	21 (18.0-25.0)	24 (20.0-31.0)	<0.001
Missing	42	40	2	
Heart rate at admission, median (IQR)	112 (94.0-131.0)	111 (94.0-130.0)	114.5 (95.5-132.0)	0.215
Missing	42	40	2	
Temperature at admission,°C, median (IQR)	36.8 (36.5-37.6)	36.9 (36.5-37.6)	36.7 (36.5-37.7)	0.439
Missing	38	36	2	
qSOFA, n (%)				<0.001
0	245 (40.7%)	197 (47.2%)	48 (25.9%)	
1	321 (53.3%)	208 (49.9%)	113 (61.1%)	
2	35 (5.8%)	12 (2.9%)	23 (12.4%)	
3	1 (0.2%)	0 (0.0%)	1 (0.5%)	
Missing	7	6	1	

*P*-values were obtained using Wilcoxon rank-sum tests for continuous variables and chi-square or Fisher’s exact tests for categorical variables, as appropriate. Missing values are shown when they are present.

Abbreviations: IQR, interquartile range; qSOFA, quick Sequential Organ Failure Assessment.

### Operational hospital impact

During the study period, the hospital evaluated approximately 900 patients with suspected measles through emergency care and epidemiological surveillance pathways. Among the 609 confirmed cases included in the analytical cohort, 186 patients required hospital admission. The outbreak also generated demand for respiratory support and higher-complexity care: 107 hospitalized patients required supplemental oxygen, seven required ICU admission, and six required invasive mechanical ventilation. In addition to direct clinical care, suspected and confirmed cases required triage, isolation procedures, laboratory coordination, epidemiological notification, and communication between the emergency, infectious diseases, pediatric, adult medicine, laboratory, and epidemiological surveillance teams. Direct financial costs and staff time were not measured prospectively and therefore could not be quantified.

### Complications, clinical management, and outcomes among hospitalized cases

Among hospitalized measles cases, pneumonia was the most frequent complication, occurring in 51 (27.4%) patients. Encephalitis was uncommon and was documented in two hospitalized patients (1.1%). More than half of the hospitalized patients required supplemental oxygen (57.5%). The maximum respiratory support was conventional oxygen in 55.4%, high-flow nasal cannula in 2.7%, and mechanical ventilation in 3.2%. ICU admission occurred in seven patients (3.8%).

Vitamin A was administered to 93 (50.3%) hospitalized patients, while systemic antibiotics were administered to nine patients (4.9%). No patients received antiviral treatment. Although pneumonia was documented in 51/186 (27.4%) hospitalized patients, systemic antibiotics were administered to nine of 186 (4.9%). This reflects that most pneumonia episodes were managed as measles-associated viral pneumonia, whereas antibiotics were used when bacterial coinfection or secondary bacterial pneumonia was clinically suspected. The median length of hospital stay was 3 days (IQR, 2-5), and two deaths were recorded among hospitalized patients, corresponding to a mortality of 1.1% within the hospitalized group (Table 2).

**Table 2:** Complications, management, and outcomes among hospitalized measles cases.

Characteristics	N = 186
Pneumonia, n (%)	
No	135 (72.6%)
Yes	51 (27.4%)
Encephalitis, n (%)	
No	184 (98.9%)
Yes	2 (1.1%)
Supplemental oxygen, n (%)	
No	79 (42.5%)
Yes	107 (57.5%)
High-flow nasal cannula, n (%)	
No	180 (96.8%)
Yes	6 (3.2%)
Mechanical ventilation, n (%)	
No	180 (96.8%)
Yes	6 (3.2%)
Maximum respiratory support, n (%)	
None	72 (38.7%)
Conventional oxygen	103 (55.4%)
High-flow nasal cannula	5 (2.7%)
Mechanical ventilation	6 (3.2%)
ICU admission, n (%)	
No	179 (96.2%)
Yes	7 (3.8%)
Vitamin A administered, n (%)	
No	92 (49.7%)
Yes	93 (50.3%)
Missing	1
Antibiotic administered, n (%)	
No	176 (95.1%)
Yes	9 (4.9%)
Missing	1
Antiviral administered, n (%)	
No	186 (100.0%)
Yes	0 (0.0%)
Length of hospital stay, days, median (IQR)	3 (2.0-5.0)
Death, n (%)	
No	184 (98.9%)
Yes	2 (1.1%)

Abbreviations: ICU, intensive care unit; IQR, interquartile range.

Among patients with documentation of at least one measles-containing vaccine dose, 12 were hospitalized and are described separately. All were pediatric patients, and all had documentation of only one vaccine dose. The median age was 5 years (IQR, 1-11), and 6/12 (50.0%) were male. All 12 cases were laboratory-confirmed and presented with fever, rash, cough, and coryza. Conjunctivitis and Koplik spots were each documented in 11/12 (91.7%) patients. Pneumonia occurred in five of 12 (41.7%) patients; 4/12 (33.3%) required supplemental oxygen; and two of 12 (16.7%) required high-flow nasal cannula. No patient in this subgroup required invasive mechanical ventilation or ICU admission, and no deaths occurred.

### Admission-associated features with hospitalization

In the epidemiological model excluding respiratory severity markers, a longer time from symptom onset to medical care was associated with hospitalization (aOR 1.14 per day; 95% CI 1.04-1.25), whereas vaccination eligibility/status showed a protective direction but was not statistically significant.

**Figure 2:**
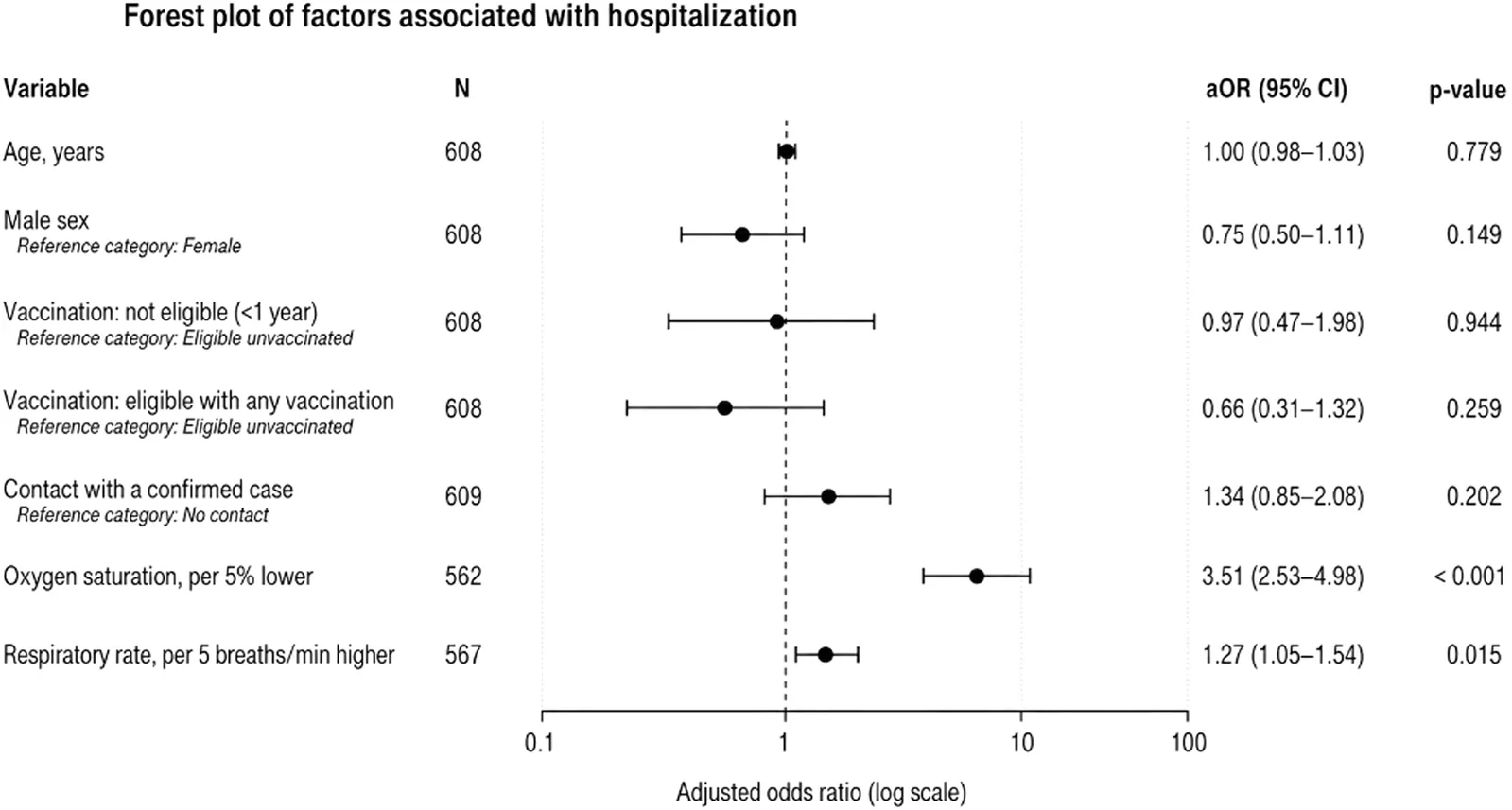
Forest plot of the clinical model among confirmed measles cases. aORs and 95% CIs were estimated using the clinical multivariable logistic regression model, which included age, sex, vaccination eligibility/status, contact with a confirmed case, oxygen saturation at presentation, and respiratory rate at presentation. Oxygen saturation was modeled per 5-percentage-point decrease, and respiratory rate was modeled per 5 breaths/min increase. The vertical dashed line indicates the null value (OR = 1). Abbreviations: aOR, adjusted odds ratio; CI, confidence interval; OR, odds ratio.

In the clinical model, objective respiratory severity markers were the main features associated with hospitalization. Each 5-percentage-point decrease in oxygen saturation was associated with higher odds of hospitalization (aOR 3.51; 95% CI 2.53-4.98), and each 5 breaths/min increase in respiratory rate was also associated with hospitalization (aOR 1.27; 95% CI 1.05-1.54) (Table 3 and Figure 2). Because of variable-specific missing data, the epidemiological model included 443 patients and the clinical presentation model included 558 patients; estimates were interpreted as complete-case analyses.

**Table 3:** Epidemiological and clinical models for hospitalization among confirmed measles cases.

Characteristics	Epidemiological model aOR (95% CI)	*P*-value	Clinical model aOR (95% CI)	*P*-value
Age, years	0.98 (0.95-1.00)	0.065	1.00 (0.98-1.03)	0.779
Male sex	0.84 (0.55-1.27)	0.402	0.75 (0.50-1.11)	0.149
Vaccination eligibility/status				
Eligible unvaccinated	Reference		Reference	
Not eligible (<1 year)	0.99 (0.47-2.02)	0.979	0.97 (0.47-1.98)	0.944
Eligible with any vaccination	0.60 (0.28-1.19)	0.159	0.66 (0.31-1.32)	0.259
Contact with confirmed case	1.35 (0.85-2.13)	0.194	1.34 (0.85-2.08)	0.202
Days from symptom onset to care	1.14 (1.04-1.25)	0.006	—	—
Oxygen saturation, per 5% lower	—	—	3.51 (2.53-4.98)	<0.001
Respiratory rate, per 5 breaths/min higher	—	—	1.27 (1.05-1.54)	0.015

**Model performance:** The epidemiological model included 443 patients and showed modest discrimination, with an AUC of 0.619 (95% CI, 0.563-0.675), no evidence of poor calibration according to the Hosmer–Lemeshow test (χ² = 7.30, df = 8, *P* = 0.505), and a Nagelkerke *R*² of 0.056. The clinical model included 558 patients and showed better discrimination, with an AUC of 0.716 (95% CI, 0.670-0.763), no evidence of poor calibration according to the Hosmer–Lemeshow test (χ² = 12.12, df = 8, *P* = 0.146), and a Nagelkerke *R*² of 0.228.

Abbreviations: aOR, adjusted odds ratio; AUC, area under the receiver operating characteristic curve; CI, confidence interval.

### Sensitivity analyses

In the alternative model using qSOFA instead of respiratory rate, lower oxygen saturation and a higher qSOFA score were independently associated with hospitalization.

In the model restricted to laboratory-confirmed cases, lower oxygen saturation and higher respiratory rate remained independently associated with hospitalization. Similar associations were observed in the pediatric-only model. In the adult-only model, a higher respiratory rate remained associated with hospitalization, whereas the association with oxygen saturation was attenuated and imprecise. An exploratory interaction analysis suggested that the association between oxygen saturation and hospitalization differed by age stratum (likelihood-ratio test *P* = 0.037), with a stronger association among pediatric patients.

In exploratory sensitivity analyses restricted to patients with valid BMI, the inclusion of BMI did not materially change the main findings. In the clinical model that included BMI, lower oxygen saturation remained strongly associated with hospitalization, whereas BMI was not associated with hospitalization. In the epidemiological model that included BMI, a longer time from symptom onset to medical care remained associated with hospitalization, while BMI again showed no association. Full results are presented in Supplementary Tables S1-S5. The clinical characteristics, management, and outcomes of hospitalized patients with confirmed measles and documented measles vaccination status are presented in Supplementary Table S6.

## Discussion

This study provides a hospital-based characterization of confirmed measles cases during a large contemporary outbreak in Jalisco, Mexico. We found that measles generated a substantial clinical burden at the referral hospital level: approximately one-third of confirmed cases required hospitalization, pneumonia was the leading complication among hospitalized patients, and more than half of the hospitalized patients required supplemental oxygen. Although most cases occurred among unvaccinated or vaccine-ineligible individuals, hospital admission was most strongly characterized by objective respiratory severity markers at presentation, especially lower oxygen saturation and a higher respiratory rate.

This study was conducted in a context of high regional transmission. By March 2026, Jalisco ranked second nationally in both confirmed measles cases and incidence rate, with transmission concentrated in municipalities within the Guadalajara metropolitan area [11]. Within this setting, our hospital served as a referral center for suspected and confirmed cases, allowing for the characterization of a large clinical cohort across pediatric and adult age groups. This is relevant because recent measles literature has often emphasized surveillance, outbreak dynamics, or pediatric hospitalized cohorts, whereas fewer studies have described hospital-based outcomes and factors associated with hospitalization in contemporary outbreaks including both children and adults [[16], [17], [18]].

The predominance of unvaccinated or vaccine-ineligible patients in our cohort is consistent with the epidemiology of the recent measles resurgence [8]. Measles remains one of the most contagious human infections, and even small reductions in vaccination coverage can permit transmission in susceptible groups. Global immunization efforts have prevented millions of deaths; however, measles continues to cause substantial morbidity and mortality, particularly among unvaccinated or undervaccinated children [19]. In addition, global coverage with the first and second doses of measles-containing vaccine remains below the threshold required to reliably interrupt transmission, reinforcing the risk of outbreaks where immunity gaps accumulate [20].

In our cohort, most confirmed cases were eligible but unvaccinated, while a relevant subgroup consisted of infants younger than one year, who were classified as not being eligible for the routine vaccination schedule. This distinction is important because vaccine-ineligible infants represent a vulnerable population that depends on herd immunity for protection. Therefore, the vaccination findings should not be interpreted only at the individual level. Although vaccination eligibility/status was not independently associated with hospitalization after adjustment for clinical severity, the high proportion of unvaccinated patients strongly suggests that insufficient population immunity contributed to the occurrence and scale of the outbreak.

The clinical presentation was largely compatible with classic measles. Fever, cough, rash, conjunctivitis, and Koplik spots were common, supporting the clinical recognition of measles during outbreak conditions. However, classic symptoms alone were not sufficient to distinguish patients who required hospitalization. Fever and rash were nearly universal, and cough produced quasi-complete separation in regression analysis because all hospitalized patients had a cough. In contrast, objective respiratory markers at presentation—oxygen saturation and respiratory rate—were more informative for identifying patients who required hospital care.

The burden among hospitalized patients was clinically meaningful. Pneumonia occurred in more than one-quarter of hospitalized cases and was the most frequent complication. This finding is consistent with previous descriptions of measles, in which pneumonia is one of the leading causes of hospitalization and measles-related mortality [21]. Recent hospital-based pediatric studies have also identified respiratory complications as a central component of measles severity, particularly in unvaccinated children and in settings experiencing outbreak resurgence [[16], [17], [18]]. Although encephalitis, ICU admission, mechanical ventilation, and death were uncommon in our cohort, their occurrence underscores the potential severity of measles during outbreaks and the need for hospital preparedness.

In the clinical model, hospitalization was primarily associated with markers of respiratory severity at presentation. Each 5-percentage-point decrease in oxygen saturation was associated with more than a threefold higher odds of hospitalization, and each 5 breaths/min increase in respiratory rate was also independently associated with hospitalization. These findings should not be interpreted as evidence that hypoxemia “causes” hospitalization. Rather, they indicate that, during a large outbreak, hospital admission was strongly aligned with objective clinical markers of respiratory compromise.

This interpretation is clinically important. In outbreak settings, emergency departments and triage areas may face a high volume of suspected cases, many of which present with similar classic symptoms. Objective parameters, such as oxygen saturation and respiratory rate, may help identify patients who require hospital-level monitoring, oxygen therapy, isolation resources, or escalation of care. This is especially relevant for hospitals serving as referral centers, where delays in recognition or inadequate triage capacity could increase both clinical risk and nosocomial transmission. Although direct financial costs and staff time were not measured, the volume of suspected cases evaluated, the number of admissions, and the need for respiratory support, isolation, laboratory confirmation, and epidemiological notification illustrate how measles outbreaks can rapidly create operational pressure in tertiary referral hospitals.

The sensitivity analyses supported the robustness of the main findings. When the analysis was restricted to laboratory-confirmed cases, lower oxygen saturation and higher respiratory rate remained independently associated with hospitalization. Similar results were observed in the pediatric-only model. In the adult-only exploratory model, respiratory rate remained associated with hospitalization, whereas the association with oxygen saturation was attenuated and less precise. The interaction analysis suggested that the association between oxygen saturation and hospitalization differed by age stratum, with a stronger association among pediatric patients. This finding should be interpreted cautiously because the adult subgroup was smaller, but it suggests that age-specific clinical thresholds and triage approaches may deserve further study.

The lack of an independent association between vaccination status and hospitalization after adjustment should be interpreted carefully. Vaccination is the main intervention to prevent measles infection, transmission, complications, and death [2]. In a hospital-based cohort restricted to confirmed cases, however, admission-associated features may reflect disease severity at presentation more than susceptibility to infection. In other words, vaccination gaps likely explain why the outbreak occurred and why so many patients became infected, while oxygen saturation and respiratory rate explain which infected patients required hospitalization.

Another consideration is the possibility of modified measles among previously vaccinated individuals. Measles in persons with pre-existing immunity may present with attenuated or atypical clinical manifestations, including milder symptoms or non-classical rash progression, which can complicate clinical recognition during outbreaks [22,23]. This phenotype may partly explain why patients with any documented vaccination showed a protective direction for hospitalization but represented a small subgroup in our cohort. However, the small number of vaccinated cases, particularly fully vaccinated individuals, limited the ability to characterize modified measles phenotypes or to compare clinical severity by dose category.

This distinction is important for public health messaging. Our findings support two complementary priorities. First, prevention remains the most effective strategy: maintaining high two-dose vaccine coverage, implementing catch-up campaigns, and protecting infants and other vulnerable groups through population immunity [20]. Second, hospitals must prepare for the clinical consequences of outbreaks that occur when these immunity gaps persist. This includes early recognition, rapid isolation, respiratory triage, availability of oxygen delivery systems, and coordination with epidemiological surveillance teams.

Measles outbreaks are not only community-level events; they also create immediate pressure on hospital systems. The need for isolation, personal protective equipment, contact tracing, laboratory confirmation, respiratory support, and dedicated clinical pathways can strain emergency departments and inpatient services [21]. In our cohort, more than half of the hospitalized patients required supplemental oxygen, and a subset required high-flow nasal cannula, mechanical ventilation, or ICU care. These findings are especially relevant for tertiary referral hospitals, where severe or complicated cases may be concentrated because of referral patterns.

The experience in Jalisco illustrates how a vaccine-preventable disease can rapidly become a hospital preparedness issue. During high-transmission periods, hospitals require operational protocols for triage, airborne isolation, protection of healthcare workers, vaccination verification of staff, and rapid communication with public health authorities. Strengthening vaccination coverage remains the cornerstone of prevention, but hospital readiness is essential for reducing morbidity and preventing secondary transmission during outbreaks.

### Strengths and limitations

This study has several strengths. It describes a large hospital-based cohort during a contemporary measles outbreak in Mexico, includes both pediatric and adult patients, uses clinical and epidemiological data from hospital and surveillance records, and evaluates admission-associated features using multivariable models and sensitivity analyses. The inclusion of laboratory-confirmed and epidemiologically linked cases reflects real-world outbreak surveillance, while the sensitivity analysis restricted to laboratory-confirmed cases supports the robustness of the main findings.

Several limitations should be acknowledged. First, this was a hospital-based study conducted at a tertiary referral center, so the findings may not represent the full spectrum of measles in the community. Referral bias is possible, particularly if more severe cases were preferentially sent to the study hospital. Second, hospitalization is a clinically relevant outcome but may be influenced by institutional criteria, bed availability, isolation capacity, and referral pathways. Third, some variables had missing data, particularly delay-related and vital sign variables, which may affect model precision. Fourth, vaccination history may have been under-documented or based on incomplete records. Fifth, although weight and height were extracted from clinical records, valid BMI could not be calculated for all patients because some height values were recorded as 0 and were treated as missing. In addition, standardized BMI-for-age classification was not performed for the full pediatric and adolescent population; therefore, BMI analyses were considered exploratory. Sixth, severe outcomes such as ICU admission, mechanical ventilation, encephalitis, and death were uncommon, limiting the ability to model these endpoints separately. We did not collect direct cost data, staff time, bed-days attributable to isolation, or detailed human-resource indicators; therefore, the financial and workforce impact of the outbreak could only be described indirectly through clinical volume, admissions, respiratory support, ICU use, and operational requirements. Seventh, the distinction between viral measles-associated pneumonia and bacterial secondary pneumonia was based on clinical documentation rather than systematic microbiological testing. Finally, although the interaction by age stratum was statistically significant, age-stratified estimates, particularly among adults, should be interpreted cautiously because of the smaller sample size.

## Conclusions

In this hospital-based cohort from a large measles outbreak in Jalisco, Mexico, measles generated a substantial hospital burden, particularly among pediatric and unvaccinated or vaccine-ineligible patients. Pneumonia was the main complication among hospitalized cases, and respiratory support was frequently required. Patients admitted to the hospital were mainly characterized by delayed medical care and objective markers of respiratory compromise at presentation, especially lower oxygen saturation and a higher respiratory rate. These findings reinforce the need for high vaccination coverage and catch-up strategies to prevent outbreaks, while also emphasizing the importance of timely medical evaluation, hospital preparedness, respiratory assessment, and rapid isolation pathways when outbreaks occur.

## CRediT authorship contribution statement

**Luis Eduardo del Moral-Trinidad:** Writing – original draft, Visualization, Methodology, Formal analysis, Data curation, Conceptualization. **Paola Elizabeth Trejo-González:** Software, Investigation, Data curation. **Christian Alberto Acosta-Aguilar:** Investigation, Data curation. **Brian Rafael Rubio-Mora:** Writing – review & editing, Validation, Supervision. **Karla Isis Avilés-Martínez:** Writing – review & editing, Supervision, Resources. **Melva Guadalupe Herrera-Godina:** Writing – review & editing, Methodology, Investigation. **Martín Guerrero-Becerra:** Writing – review & editing, Resources. **Rodrigo Escobedo-Sánchez:** Writing – review & editing, Supervision, Software, Resources. **Esteban González-Díaz:** Writing – review & editing, Project administration. **Gilberto Silva-Bañuelos:** Writing – original draft, Supervision, Methodology, Formal analysis, Conceptualization.

## Declaration of competing interest

The authors have no competing interests to declare.

## Appendix Supplementary materials

Image, application 1
