# Supplementary material for: Hospital burden and clinical features associated with admission among confirmed measles cases during the 2025-2026 outbreak at a tertiary care hospital in Mexico
Source: IJID Reg. 2026 Jul 31;20:100965. doi: 10.1016/j.ijregi.2026.100965 (PMC13571253; doi:10.1016/j.ijregi.2026.100965)
Supplement: Supplementary file 1 [file mmc1.docx]

**Supplementary Table S1. Univariable logistic regression for hospitalization among measles cases**

| Characteristic | N | OR (95% CI) | p-value |
| --- | --- | --- | --- |
| Age, years | 608 | 0.98 (0.96–0.99) | 0.006 |
| Age stratum | 609 |  |  |
| Pediatric |  | Reference |  |
| Adult |  | 0.75 (0.49–1.13) | 0.184 |
| Sex | 608 |  |  |
| Female |  | Reference |  |
| Male |  | 0.85 (0.60–1.20) | 0.352 |
| Vaccination eligibility/status | 608 |  |  |
| Eligible unvaccinated |  | Reference |  |
| Not eligible (<1 year) |  | 1.47 (0.83–2.55) | 0.180 |
| Eligible with any vaccination |  | 0.54 (0.27–1.00) | 0.063 |
| Travel in the previous 2 weeks | 609 |  |  |
| No |  | Reference |  |
| Yes |  | 0.48 (0.11–1.49) | 0.252 |
| Contact with a confirmed case | 609 |  |  |
| No |  | Reference |  |
| Yes |  | 1.43 (0.97–2.10) | 0.071 |
| Final case classification | 609 |  |  |
| Laboratory-confirmed |  | Reference |  |
| Epidemiologically linked |  | 0.81 (0.35–1.71) | 0.596 |
| Days from symptom onset to medical care | 444 | 1.15 (1.05–1.26) | 0.003 |
| Immunosuppression | 609 |  |  |
| No |  | Reference |  |
| Yes |  | 1.14 (0.05–11.9) | 0.916 |
| Number of comorbidities | 609 | 1.23 (0.40–3.39) | 0.692 |
| Conjunctivitis | 608 |  |  |
| No |  | Reference |  |
| Yes |  | 1.91 (1.28–2.91) | 0.002 |
| Koplik spots | 609 |  |  |
| No |  | Reference |  |
| Yes |  | 1.70 (1.18–2.45) | 0.004 |
| Oxygen saturation, per 5% lower | 562 | 3.86 (2.82–5.43) | <0.001 |
| Respiratory rate, per 5 breaths/min higher | 567 | 1.42 (1.23–1.64) | <0.001 |
| Temperature at admission, °C | 571 | 0.96 (0.78–1.18) | 0.713 |
| qSOFA | 602 | 2.55 (1.87–3.50) | <0.001 |

**Note:** Cough was not included in the univariable logistic regression table because all hospitalized patients had cough, leading to quasi-complete separation and unstable estimates.

**Supplementary Table S2. Clinical model additionally adjusted for delay to care**

| Characteristic | Adjusted OR (95% CI) | p-value |
| --- | --- | --- |
| Age, years | 1.01 (0.98–1.04) | 0.660 |
| Male sex | 0.78 (0.48–1.25) | 0.301 |
| Vaccination eligibility/status |  |  |
| Eligible unvaccinated | Reference |  |
| Not eligible (<1 year) | 0.82 (0.34–1.93) | 0.652 |
| Eligible with any vaccination | 0.72 (0.32–1.49) | 0.391 |
| Contact with a confirmed case | 1.34 (0.79–2.24) | 0.268 |
| Days from symptom onset to care | 1.11 (1.00–1.23) | 0.047 |
| Oxygen saturation, per 5% lower | 3.34 (2.32–4.98) | <0.001 |
| Respiratory rate, per 5 breaths/min higher | 1.30 (1.03–1.64) | 0.026 |

**Model performance:** Hosmer–Lemeshow p = 0.308; AUC = 0.737 (95% CI 0.683–0.791); Nagelkerke R² = 0.249.

**Abbreviations:** OR, odds ratio; CI, confidence interval; qSOFA, quick Sequential Organ Failure Assessment.

**Supplementary Table S3. Alternative model using qSOFA**

| Characteristic | Adjusted OR (95% CI) | p-value |
| --- | --- | --- |
| Age, years | 1.00 (0.98–1.03) | 0.872 |
| Male sex | 0.74 (0.50–1.10) | 0.142 |
| Vaccination eligibility/status |  |  |
| Eligible unvaccinated | Reference |  |
| Not eligible (<1 year) | 1.32 (0.65–2.68) | 0.436 |
| Eligible with any vaccination | 0.64 (0.30–1.28) | 0.227 |
| Contact with a confirmed case | 1.49 (0.95–2.34) | 0.081 |
| Oxygen saturation, per 5% lower | 3.61 (2.58–5.17) | <0.001 |
| qSOFA | 1.80 (1.23–2.65) | 0.003 |

**Model performance:** Hosmer–Lemeshow p = 0.293; AUC = 0.730 (95% CI 0.685–0.776); Nagelkerke R² = 0.242.

**Supplementary Table S4. Additional sensitivity and stratified analyses for hospitalization among measles cases**

| Characteristic | Laboratory-confirmed cases only, aOR (95% CI) | p-value | Pediatric-only model, aOR (95% CI) | p-value | Adult-only model, aOR (95% CI) | p-value |
| --- | --- | --- | --- | --- | --- | --- |
| Age, years | 1.01 (0.98–1.03) | 0.601 | 1.04 (0.98–1.10) | 0.216 | **0.88 (0.80–0.95)** | **0.002** |
| Sex |  |  |  |  |  |  |
| Female | Reference |  | Reference |  | Reference |  |
| Male | 0.77 (0.52–1.16) | 0.216 | 0.86 (0.55–1.36) | 0.531 | **0.39 (0.15–0.96)** | **0.048** |
| Vaccination eligibility/status |  |  |  |  | — | — |
| Eligible unvaccinated | Reference |  | Reference |  | — | — |
| Not eligible (<1 year) | 0.97 (0.46–2.01) | 0.937 | 1.32 (0.60–2.88) | 0.490 | — | — |
| Eligible with any vaccination | 0.71 (0.34–1.43) | 0.361 | 1.15 (0.51–2.45) | 0.724 | — | — |
| Contact with a confirmed case |  |  |  |  | — | — |
| No | Reference |  | Reference |  | — | — |
| Yes | 1.26 (0.80–1.99) | 0.315 | 1.30 (0.78–2.16) | 0.313 | — | — |
| Oxygen saturation, per 5% lower | **3.55 (2.54–5.09)** | **<0.001** | **4.16 (2.87–6.27)** | **<0.001** | 1.73 (0.84–3.84) | 0.149 |
| Respiratory rate, per 5 breaths/min higher | **1.26 (1.03–1.53)** | **0.024** | **1.33 (1.07–1.65)** | **0.010** | **3.32 (1.36–9.04)** | **0.013** |

**Interaction analysis:** In an exploratory interaction model, the association between oxygen saturation and hospitalization differed by age stratum (likelihood-ratio test p=0.037). The estimated association for each 5-percentage-point decrease in oxygen saturation was stronger among pediatric patients (OR, 4.21) than among adults (OR, 1.76).

**Notes:** The adult-only model was fitted with a more parsimonious covariate set because of the smaller number of adult hospitalizations. Vaccination eligibility/status and contact with a confirmed case were not included in the adult-only model.
**Abbreviations:** aOR, adjusted odds ratio; CI, confidence interval; OR, odds ratio.

**Supplementary Table S5. Exploratory BMI sensitivity analyses for hospitalization among confirmed measles cases.**

| Characteristic | Clinical model + BMI, aOR (95% CI) | p-value | Epidemiological model + BMI, aOR (95% CI) | p-value |
| --- | --- | --- | --- | --- |
| Age, years | 0.98 (0.95–1.01) | 0.284 | 0.97 (0.94–1.01) | 0.120 |
| Male sex | 0.74 (0.47–1.15) | 0.184 | 0.81 (0.51–1.30) | 0.393 |
| Not eligible (<1 year) | 0.87 (0.37–1.99) | 0.751 | 1.05 (0.44–2.44) | 0.907 |
| Eligible with any vaccination | 0.76 (0.34–1.60) | 0.490 | 0.66 (0.29–1.39) | 0.296 |
| Contact with confirmed case | 1.37 (0.83–2.26) | 0.216 | 1.42 (0.85–2.37) | 0.179 |
| Days from symptom onset to care | — | — | 1.12 (1.01–1.25) | 0.038 |
| Oxygen saturation, per 5% lower | 3.78 (2.60–5.68) | <0.001 | — | — |
| Respiratory rate, per 5 breaths/min higher | 1.21 (0.97–1.50) | 0.092 | — | — |
| BMI, kg/m² | 1.02 (0.98–1.07) | 0.309 | 1.00 (0.95–1.05) | 0.949 |

Note: The clinical model including BMI included 444 patients. The epidemiological model including BMI included 341 patients. BMI analyses were exploratory and restricted to patients with valid height and weight data. Height values recorded as 0 were treated as missing. Because BMI interpretation differs by age and requires age- and sex-specific references in children and adolescents, BMI was evaluated as a continuous variable and was not included in the primary models. Abbreviations: aOR, adjusted odds ratio; BMI, body mass index; CI, confidence interval.

**Supplementary Table S6. Clinical characteristics, management, and outcomes of hospitalized confirmed measles cases with documented measles vaccination****

| Characteristic | N = 12^1^ |
| --- | --- |
| Age, years | 5.0 (1.0–11.0) |
| Pediatric age (<18 years) | 12 (100.0%) |
| Sex |  |
| Female | 6 (50%) |
| Male | 6 (50%) |
| Number of documented vaccine doses |  |
| None | 0 (0.0%) |
| One dose | 12 (100.0%) |
| Two doses | 0 (0.0%) |
| Booster | 0 (0.0%) |
| Unknown | 0 (0.0%) |
| Documented vaccination status |  |
| Unvaccinated | 0 (0.0%) |
| Partial | 12 (100.0%) |
| Complete | 0 (0.0%) |
| Unknown | 0 (0.0%) |
| Final case classification |  |
| Laboratory-confirmed | 12 (100.0%) |
| Epidemiologically linked | 0 (0.0%) |
| Days from symptom onset to medical care | 3.5 (2.0–5.0) |
| Fever | 12 (100.0%) |
| Rash | 12 (100.0%) |
| Cough | 12 (100.0%) |
| Coryza | 12 (100.0%) |
| Conjunctivitis | 11 (84.6%) |
| Koplik spots | 11 (84.6%) |
| Oxygen saturation at presentation, % | 94.0 (91.0–96.0) |
| Respiratory rate at presentation | 26.0 (21.0–30.0) |
| Pneumonia | 5 (38.5%) |
| Encephalitis | 0 (0.0%) |
| Supplemental oxygen | 4 (30.8%) |
| High-flow nasal cannula | 2 (15.4%) |
| Mechanical ventilation | 0 (0.0%) |
| ICU admission | 0 (0.0%) |
| Vitamin A administered | 8 (66.7%) |
| Missing | 1 |
| Systemic antibiotics | 0 (0.0%) |
| Length of hospital stay, days | 4.0 (2.0–7.0) |
| Death | 0 (0.0%) |
| ^1^Median (Q1–Q3); n (%) | |
